# Various Substitute Aggregate Materials for Sustainable Concrete

**DOI:** 10.3390/ma15238658

**Published:** 2022-12-05

**Authors:** Se-Jin Choi, Jae-Eun Oh, Se-Yoon Yoon

**Affiliations:** 1 Department of Architectural Engineering, Wonkwang University, Iksan 54538, Republic of Korea; 2 School of Urban and Environmental Engineering, Ulsan National Institute of Science and Technoloty, Ulsan 44919, Republic of Korea; 3 Department of Civil Engineering, Kyonggi University, Suwon-si 16227, Republic of Korea

Concrete is one of the most widely used structural construction materials and has significantly influenced industrial development. Recently, various efforts have been made to reduce greenhouse gases worldwide, with the concrete industry also making efforts to move toward sustainable development. In particular, owing to the immense growth of the global concrete industry, the shortage of natural aggregates has become a serious problem. Therefore, a considerable amount of social and research interest has focused on finding alternative aggregate materials to replace natural aggregates. Considering that aggregates comprise a large proportion of the concrete volume, they significantly influence the mechanical properties and durability of the concrete. Therefore, when replacing the existing natural aggregate with a substitute aggregate material, there should be no deterioration in the performance of the concrete; in this way, sustainable concrete development can be achieved.

From this perspective, this Special Issue is a collection of ten papers focused on various alternative aggregate materials for sustainable concrete development, including substitute aggregate materials, artificial aggregates, recycled aggregates, and functional concrete materials. In the Editors’ opinion, each article presents a novel, clear, scientific idea from various standpoints (analytical, experimental, and conceptual), thus representing a major contribution to our understanding the mechanical behavior of various aggregates and concrete materials. The Editors hope this article collection can contribute to the continuous research for a more thorough and reliable understanding of the mechanical behavior or durability characteristics of sustainable aggregates and concrete materials.

Among the experimental contributions, Wenhao et al. [1] evaluated the effect of lightweight, functional aggregates on mitigating the anode degradation of the impressed current cathodic protection of reinforced concrete. Studies have shown that the presence of these functional aggregates help mitigate acidification propagation and distribute a protective current more uniformly, thus demonstrating the importance and effectiveness of acidification inhibition for anode performance optimization. Lucyna et al. [2] reviewed the effect that impregnating lightweight aggregates (LWAs) with cement paste had the aggregates’ properties and verified their effectiveness in concrete. The assessment of the properties of LWAs precoated with cement paste and their suitability for concrete showed the purposefulness of applying such a treatment to reduce the water content in fresh concrete and how it is necessary to maintain target workability as well as tighten and strengthen hardened concrete.

Se-Jin et al. [3] synthesized catechol-functionalized chitosan (Cat-Chit), a well-known bioinspired polymer, and used it to evaluate the properties of cement mortar. The sample containing 7.5% Cat-Chit polymer in water (CPW) exhibited the highest compressive strength, as its 28-day compressive strength was ~20.2% higher than that of the control sample with no added polymer.

Sung-Ho et al. [4] investigated how the properties of mortar containing a steel slag aggregate affected its performance, analyzing its fluidity, compressive strength, tensile strength, accelerated carbonization depth, chloride ion penetration resistance, and the presence or absence of blast furnace slag powder (BFSP). Studies have shown that using an appropriate combination of BFSP and a steel slag aggregate can significantly increase the replacement rate of steel slag as an aggregate.

Maria et al. [5] examined the applicability of waste foundry exhaust sand (WFES) in self-compacting concrete (SCC) and reported that the compressive strength and sulfate resistance of SCC increased when replaced with 30% of WFES. They suggested that this can contribute to the development of sustainable concrete as it helps reduce the use of cement and natural aggregates and decreases the volume of waste in landfills.

Ji-Hwan et al. [6] applied new concrete reinforcing fibers, an amorphous metallic fiber (AMF), and a steel slag aggregate to mortar and evaluated the various mechanical properties of the mortar. The testing results revealed that the 28-day compressive strength of the sample with the steel slag aggregate and AMFs was between 48.7 and 50.8 MPa, which is equivalent to or higher than that of the control sample (48.7 MPa). The AMFs had a remarkable effect on improving the tensile strength of the mortar, regardless of the use of natural aggregates.

In two papers, 3D-printed capsules were researched. Taeuk et al. [7] produced specimens in accordance with ASTM specifications using the FDM-PLA method, with their mechanical properties obtained through tensile, shear, and compression tests. As a result, the newly proposed capsule design was verified to have an isotropic fracture strength of 1400% in all directions compared with conventional spherical thin-film capsules. In addition, Se-Jin et al. [8] evaluated the mechanical properties and solubility of 3D-printed capsules in self-healing cement composites. Their findings suggest that a thicker capsule wall can withstand a larger bursting load, whereas the rupture characteristics varied depending on the printing angle. These results can be used to optimize the capsule design parameters for different cement environments.

Tianyu et al. [9] examined the durability of high-performance, fiber-reinforced mortar in a simulated wastewater pipeline environment and obtained the following conclusions: the high-performance, fiber-reinforced mortar (M) group had a better visual appearance than the ordinary mortar (P) group after corrosive cycles, and the height loss of the M group was less than 80% of that of the P group after 12 corrosion cycles, indicating that the M group had better dimensional stability in corrosive environments.

Finally, Se-Jin et al. [10] evaluated the durability of cement composites using recycled water (RW) and a blast furnace slag aggregate (BSFA) and reported that the 28-day compressive strengths of all samples using the RW and BSFA were higher than those of the control sample. They suggested that when the RW and BSFA are used properly, the mechanical properties of the cement mortar, carbonation resistance, and chloride ion penetration resistance are expected to be effectively improved.

## Data Availability

Not applicable.
